# Screening of anti-inflammatory and antipyretic activity of *Vitex leucoxylon* Linn

**DOI:** 10.4103/0253-7613.71891

**Published:** 2010-12

**Authors:** Padmini Shukla, P. Shukla, S.B. Mishra, B. Gopalakrishna

**Affiliations:** Pranveer Singh Institute of Technology, Kanpur, India; 1 Roorki College of Pharmacy, Roorki, Uttar Pradesh, India; 2 R.R. College of Pharmacy, Chikbanawara, Bangalore, India

**Keywords:** Inflammation, pyrexia, *Vitex leucoxylon*

## Abstract

**Objective:**

This study was designed to evaluate the anti-inflammatory and antipyretic activity of ethyl acetate extract of *Vitex leucoxylon* Linn. in various animal experimental models.

**Materials and Methods:**

Ethyl acetate extract of *V. leucoxylon* Linn. evaluated for anti-inflammatory activity in carrageenan, mediator-induced rat paw edema, and cotton pellet-induced granuloma model. The antipyretic activity was evaluated by yeast-induced pyrexia model.

**Results:**

Single administration of the ethyl acetate extract of *V. leucoxylon* Linn. at dose of 500 mg/kg p.o. showed significant (*P* < 0.001) inhibition of rat paw edema. The ethyl acetate extract showed significant antipyretic activity in brewer yeast-induced pyrexia in rats throughout the observation period of 4 h.

**Conclusion:**

This study shows that ethyl acetate extract of *V. leucoxylon* Linn. has significant anti-inflammatory and antipyretic activity.

## Introduction

Plants belonging to genus Vitex (Fam—Verbenaceae) are therapeutically important in ayurvedic material medica. *Vitex leucoxylon* Linn. is a small-to-large tree with a short-thick trunk and a spreading crown found almost throughout Deccan Peninsula. The root and bark are astringent, and the root is used as a febrifuge. The leaves are smoked for relieving headache, and catarrh are also used for medicinal baths in fever and anemia.[1] The bark of *V. leucoxylon* mainly contains β-sitosterol, vitexin, dimethyl terephthalate, isovitexin, and other flavonoids. It is reported that the crude aqueous extract has anti-inflammatory and wound-healing activities.[2] The aim of the study was to ascertain the anti-inflammatory and antipyretic properties of this plant.

## Materials and Methods

### Collection and preparation of extract

The bark of *V. leucoxylon* Linn were collected from the bank of the pond known as Chennapur (Canara circle, Sirsi division) in Bachagaon village, authenticated by Prof. and Dr. B.D. Huddar, Head, Department of Botany, HSK College of Arts and Sciences, Hubli, Karnataka. For the preparation of ethyl acetate extract, the shade dried powdered leaves (100 gm) were extracted with ethyl acetate using soxhlet assembly. The extract was concentrated in a rotary flash evaporator under reduced pressure to semi-solid mass. The residue was dried in a desiccator over sodium sulfite (yield = 13.5).

The phytochemical screening of EAVL was done according to the methods described by Kokate *et al*.[3]

### Animals

Colony bred healthy Wistar albino rats of either sex weighing 150–200 g were used. They were housed in standard conditions exposed to 12:12 h, light:dark cycle. They were fed standard diet and water *ad libitum*. The Ethics Committee KLE’S College of Pharmacy, Hubli, approved the protocol of the study (CPCSEA Registration no. is 126/1999/CPCSEA).

## Anti-inflammatory Activity

### Carrageenan-induced paw edema

The rats were divided into three groups of six animals each. Acute inflammation was induced by intraplantar administration of 0.1 mL of carrageenan (1% solution in normal saline). Group A was treated with vehicle (10 mL/kg p.o.), Group B with indomethacin (10 mg/kg p.o.), and Group C with EAVL (500 mg/kg p.o.) 1 h before administration of phlogistic agent. The paw volume was measured prior to injection of phlogistic agent (0 h) and then at a predetermined interval of 60 min up to 3 h after carrageenan injection. Paw volume was measured using Digital Plethysmometer (UGO Basil, Italy). Change in the paw volume was measured, and anti-inflammatory activity was calculated as follows:

% Inhibition of inflammation = 1 - (Vt/Vc) × 100,

where Vt represents the change in the paw volume in EAVL-treated group and Vc represents the change in the paw volume in the corresponding vehicle-treated control group.

### Serotonin-induced paw edema

In serotonin-induced paw edema model, the same procedure was carried out, except that 0.1 mL of serotonin (1%) was injected instead of carrageenan.

### Cotton pellet-induced granuloma

The rats were divided into three groups (*n* = 6). After shaving the fur, the rats were anesthetized with ether and 20 mg of sterile cotton pellets was surgically inserted in the groin region. The EAVL (500 mg/kg, p.o.), indomethacin (10 mg/kg, p.o.), and vehicle (10 mL/kg p.o.) were administered to Group C, Group B, and Group A, respectively, for seven consecutive days from the day of cotton pellet implantation. The animals were anesthetized on the 8^th^ day, and the cotton pellets were removed surgically and made free from extraneous tissues. The pellets were incubated at 37°C for 24 h and dried at 60°C for constant weight. Increment in the dry weight of the pellets was taken as measure of granuloma formation.[4]

## Antipyretic Activity

### Brewer’s Yeast Pyrexia model

Antipyretic activity on albino rats was studied with fever induced by 20% Brewer yeast. Albino rats (150–200 gm) were fed uniformly till 21 hr and after measuring rectal temperature of the animals by introducing 1.5 cm of digital thermometer in rectum. Pyrexia was induced by injecting 20% suspension of dried yeast in 2% gum acacia in normal saline at a dose of 20 mL/kg of body weight subcutaneously. After 18 h of yeast injection, rats which showed a rise in temperature of at least 1°F (0.6°C) were taken for the study. The rats were then divided into three groups (*n* = 6). Group A received vehicle (3% gum acacia suspension 1 mL/200 g p.o.), Group B received standard drug aspirin (300 mg/kg p.o.), and Group C received EAVL (500 mg/kg p.o.). The rectal temp was recorded every hour for 4 h after administration of drugs.[5]

### Statistical analysis

The results are presented as mean ± SEM. Statistical analysis of data was performed using Student’s *‘t’*-test to study the differences among the means.

## Results

Phytochemical screening indicates the presence of flavonoids and polyphenolic constituents. As shown in Table 1, ethyl acetate extract of *V. leucoxylon* exhibited inhibition in various models of inflammation. Table 2 shows the effect of *V. leucoxylon* in yeast-induced pyretic rats. There was no significant difference in the basal temperature at ‘0’ h between the different groups. However, the ethyl acetate extract of *V. leucoxylon* and aspirin significantly decreased the temperature of pyretic rats at second, third, and fourth hours after drug or extract treatment.

**Table 1:** Anti-inflammatory effect of EAVL in various models of inflammation

*Groups, n = 6*	*Dose (mg/kg)*	*Carrageenan-induced model*			*Serotonin-induced model*			*Cotton pellet-induced model*
		*1h*	*2h*	*3h*	*1 h*	*2h*	*3h*	*Increased weight of dry cotton pellet (mg)*
		*EV (mL)*	*EV (mL)*	*EV (mL)*	*EV (mL)*	*EV (mL)*	*EV (mL)*	
Control		0.61 ± 0.003	0.68 ± 0.023	0.79 ± 0.015	0.61 ± 0.003	0.61 ± 0.026	0.69 ± 0.02	38.61 ± 0.10
Indomethacin	10	0.52 ± 0.012 (14.7%)	0.50 ± 0.013* (29.75%)	0.49 ± 0.083* (37.9%)	0.52 ± 0.012 (15.6%)	0.49 ± 0.012 (29.4%)	0.47 ± 0.76* (38.4%)	18.71 ± 0.44* (50.54%)
EAVL	500	0.56 ± 0.005 (8.19%)	0.49 ± 0.014* (26.5%)	0.50 ± 0.008* (36.71%)	0.56 ± 0.005 (9.7%)	0.5 ± 0.014 (27.8)	0.56 ± 0.008* (37.6%)	23.31 ± 0.21* (39.67%)

* Significant at *P* < 0.001, P-value was calculated by comparing with control by ANOVA followed by the Student t-test, Values are expressed as mean ± SEM.

**Table 2:** Anti-pyretic effect of EAVL on brewer’ s yeast-induced pyrexia in rats

*Group*	*Dose*	*Rectal temperature in °C at time (min)*					
		*-18^a^*	*0 min*	*60 min*	*120 min*	*180 min*	*240 min*
Control		36.9 ± 0.036	37.6 ± 0.022	37.65 ± 0.022	38.25 ± 0.022	38.35 ± 0.022	39.25 ± 0.022
EAVL	500 mg/kg	37.5 ± 0.10	38.4 ± 0.15*	37.1 ± 0.12*	36.78 ± 0.052*	36.72 ± 0.047	36.7 ± 0.054*
Aspirin	300 mg/kg	37.5 ± 0.05	38.17 ± 0.02*	36.8 ± 0.042*	36.59 ± 0.042*	36.6 ± 0.062	36.4 ± 0.062*

*n* = 6. Values are mean ± SEM,

* *P* < 0.001 when compared to control;

a Temperature just before yeast injection

b Temperature just after yeast injection

## Discussion

Variety of indigenous drugs are used for relief in inflammation. The most widely used primary test to screen new anti-inflammatory agents measures the ability of a compound to reduce local edema induced in the rat paw by an injection of an irritant agent.[6] This edema depends on the participation of kinins and polymorphonuclear leucocytes with their pro-inflammatory factors including prostaglandins.[7] The development of edema in the paw of the rats after the injection of carrageenan has been described as a biphasic event. The initial phase, observed around 1 h, is attributed to the release of histamine and serotonin; the second accelerating phase of swelling is due to the release of prostaglandin-like substances.

It has been reported that the second phase of edema is sensitive to both clinically useful steroidal and nonsteroidal anti-inflammatory agents. Significant anti-inflammatory activity was observed for ethyl acetate extract of *V. leucoxylon* in carrageenan- and serotonin-induced edema model. Since prostaglandins are involved in swelling and are inhibited by flavonoids,[8] it could be suggested that reduced availability of prostaglandins by flavonoids of EAVL might be responsible for its anti-inflammatory effect.

It is well known that most of the anti-inflammatory analgesic drugs possess antipyretic activity. EAVL revealed marked antipyretic activity in brewers yeast-induced rats. In general, nonsteroidal anti-inflammatory drugs produce their antipyretic action through inhibition of prostaglandin synthesis within the hypothalamus.[9] The antipyretic effect of the test drug may be due to presence of flavonoid compounds, as some flavonoids are predominant inhibitors of cyclooxygenase or lipoxygenase.

In conclusion, this study demonstrates that EAVL has marked antipyretic and moderate anti-inflammatory activities.
